# Chemometric modeling of odor threshold property of diverse aroma components of wine†

**DOI:** 10.1039/c7ra12295k

**Published:** 2018-01-25

**Authors:** Probir Kumar Ojha, Kunal Roy

**Affiliations:** Drug Theoretics and Cheminformatics Laboratory, Department of Pharmaceutical Technology, Jadavpur University Kolkata 700 032 India kunalroy_in@yahoo.com kunal.roy@jadavpuruniversity.in http://sites.google.com/site/kunalroyindia/ +91-33-2837-1078 +91-98315-94140

## Abstract

We have modelled here odor threshold properties (OTP) of various aroma components present in different types of wine using quantitative structure–property relationship (QSPR) studies employing both two-dimensional and three-dimensional descriptors. The aim has been to identify the molecular properties essential for lowering the OTP. We have applied different variable selection strategies to select the most relevant descriptors prior to the development of the final partial least squares (PLS) regression model, which was validated extensively using different validation metrics in terms of acceptability and predictivity of the model for enhancing confidence in QSPR predictions. Using the developed PLS model, we have also predicted the “composite” OTP of different types of wine using the “composite” descriptor values based on individual components according to the PLS model and the results were well corroborated with the observations reported by Wang *et al.* [*Food Chem.*, 2017, **226**, 41–50]. The developed model may guide us to understand the dependence of the odor quality of different types of wines obtained under different manufacturing conditions on their aroma constituents.

## Abstract 2

We have modelled here odor threshold properties (OTP) of various aroma components present in different types of wine using quantitative structure–property relationship (QSPR) studies employing both two-dimensional and three-dimensional descriptors.

## Graphical abstract

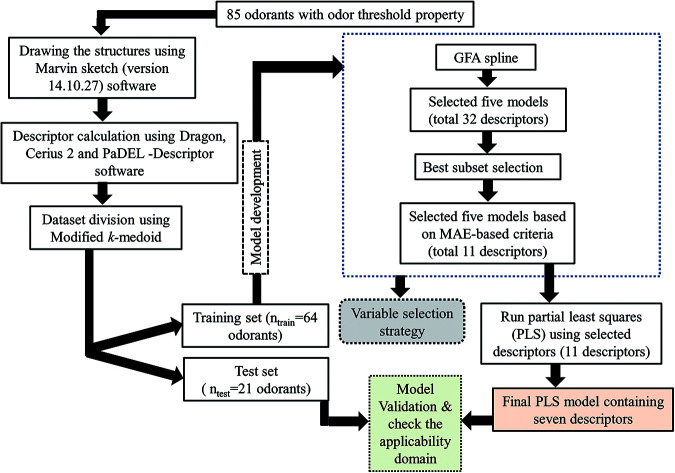

## 1. Introduction

Fragrance chemistry is a very charming area for researchers since it has a very strong relationship with our day to day life. The molecules relevant to fragrance chemistry impart both odor and flavour.^2^ Often, olfaction in humans is totally underestimated or overlooked. The sense of smell is usually supposed to play only a negligible role in human perceptual experiences, which is a misapprehension when taking into account the daily dominance of vision and hearing for communication.^3^ Nowadays, odorant molecules are widely used in consumer products like pharmaceuticals, cosmetics, food and beverages, paints and varnishes, detergents, fabric softeners, cleaning products, perfumes, skin creams *etc.*^4^ The odorant molecules make a pleasant sense to the products obtained from these sectors. Even, the unpleasant odors of various chemicals present in different formulations or products can be masked by using pleasant odorants molecules as adjuvant. Thus, olfaction has emerged as a prime topic of interest for researchers for many decades. In this perspective, it might be useful to define what an odor and odor threshold are. An odor is the notion in the brain elicited by the recognition of a (mostly) volatile chemical compounds at a very low concentration by an odorant receptor that is perceived by the sense of olfaction of human or other animals. The odor threshold is determined in a fixed volume odor test room containing low background odor for presenting the stimulus of the observer. Trained odor analysts are used to identify the odor threshold. The odor response is defined rigorously by requiring each panel member to describe the odor quality. Each panel member defines the odor response and describes the odor quality. A pool of approximately fifteen observers with more than one year of analytical odor work might be selected as panel members.^5^ The odor threshold (OT) is the minimum concentration at which entire panel members have been capable to identify the odor perception which is a typical characteristic of that individual chemical and have been consistent in their response at all higher test concentrations. A chemical can be considered as an odor active molecule if it fulfils the criteria like (i) it binds to an odorant receptor; (ii) the odorant receptor transmits the recognition to the brain and (iii) the brain recognizes it as a signal that can be interpreted.

Wine is one of the most important beverages where odorants play a crucial role to distinguish the quality among the different types of wines. Wine contains various types of odorants like phenols, alcohols, esters, fatty acids, terpenols, furans, thiols, lactones *etc.* with different concentrations. The presence of aroma components and their concentration in wine depends on the way of manufacturing. Noble rot wine is a sweet wine prepared by botrytizing the grapes with a beneficial fungus *B. cinerea*, also called noble rot. The botrytization of grapes undergoes different enzymatic reaction and dehydration of the grape berry resulting in a higher concentration of sugars, acids, glycerol, minerals, and certain aroma components.^6^ It has been found that noble rot wine from Bordeaux Sauternes contains higher levels of volatile aroma components including homofuraneol, furaneol, norfuraneol, phenyl-acetaldehyde and methional than un-botrytized dry white wine.^7^ The actual mechanism behind human olfaction is very complex, and it has been assumed that it is mediated through G-protein coupled receptors lining the nasal epithelium.^8^ Since there is no such modern technology which can actually mimic the efficiency of human nose and can characterize different types of odor with the similar sensitivity, it is very difficult to identify the structural properties which are essential for odor threshold property of wines. In this perspective, chemometric modelling *via* quantitative structure–activity/property relationship (QSAR/QSPR)^9–12^ approach may help us to study the correlation between molecular properties and odor threshold properties. QSAR attempts to correlate the structural/molecular properties in the form of descriptors with biological activities/properties/toxicities for a set of compounds by using different chemometric tools. In the past, some researchers have reported QSPR models of components present in wine.^13,14^ Duchowicz *et al.*^13^ developed linear regression equations for the amino acid concentration profile present in Merlot and Torrontes wine. In 2009, Rastija *et al.*^14^ reported QSPR equations for various physicochemical properties of ployphenol class of compounds (19 polyphenols) present in wine (limited applicability domain). However, these studies did not consider modeling OTP of wine components. Again, although there are some reports of QSPR modelling of OTP of some classes of odorants in general (for example, pyrazine derivatives,^15^ aliphatic alcohols,^16^ diverse air-borne volatile organic compounds^17^), there has been no attempt so far to model specifically the odorant components of wine. Such attempt will definitely help to understand the dependence of the odor quality of different types of wines on their aroma constituents (for example, different manufacturing conditions may lead to different composition of odorant constituents in wine leading to a difference in odor quality). Here, we have developed a QSPR model using diverse classes of odorants (85 odorants providing a wide applicability domain) present in different types of wines.

In this paper, we have developed a QSPR model using various aroma components present in different types of wine to identify the molecular properties essential for odor threshold property. The methodologies employed in this study for the development of predictive QSPR models are in accordance to the principles of QSAR model development recommended by Organization for Economic Co-operation and Development (OECD) (http://www.oecd.org/chemicalsafety/risk-assessment/37849783.pdf). Here, we have employed a variable selection approach to down size the descriptor pool prior to development of final model in order to avoid the chance of overfitting and get rid of noise or redundant information. Previously also, many researchers reported different strategies for selection of descriptors prior to development of final QSAR/QSPR models.^18–22^ Note that, while developing the models, we have kept aside some compounds as hold out samples (test set) which have not been used for the model development. After the model has been developed, the predictive quality of the developed model has been evaluated based on the experimental values of the hold out samples. This is a standard process of evaluating the predictive potential of QSPR models according to the OECD guidelines. In this work, we have also predicted the composite odor threshold property of different types of wine using the information obtained from the developed PLS model.

## 2. Materials and methods

### 2.1 Dataset

The present work deals with QSPR modeling of diverse classes of 85 aroma producing compounds with defined odor threshold property and present in wines collected from two sources.^1,23^ Among the odorant molecules reported in these two references, 25 molecules are common. The reported odor threshold property values of seventeen out of twenty five odorants in the two source references are exactly same. This proves the homogeneity in the data sets and similarity in experimental protocols in the two original sources enabling us a merger of the two sets. The combined dataset comprises higher alcohols, esters, fatty acids, C6 compounds, terpenols, C13-norisoprenoids, furans, thiols, lactones, phenylethyls, phenolic acid derivatives and volatile phenols. For aroma component analysis, solid-phase micro-extraction gas chromatography–mass spectrometry (SPME-GC–MS) was used.^24^ The original authors have performed the sensory analysis by a trained tasting panel of 30 students.^1^ The panel had been trained with a Le Nez du Vin standard aroma kit based on the procedures of Tao *et al.*^25^ The standard ‘‘Le Nez du Vin” aroma kit was made up with 54 vials, where each and every vial contained one characteristics aroma property in wine like Green pepper, Blackcurrant, Prune, Smoke, Cut hay, Mint, Violet, *etc.* There was an aroma identification test every weekend after the training of panel members thrice a week for 60–90 min. The ability of the panelists to discriminate the aroma property and measurement of odor threshold was carried out before and after the training. Four reference compounds with different aromas were dissolved in synthetic wines contained 11% (v/v) alcohol, 6 g L^−1^ of tartaric acid, and added 1 M NaOH for maintaining the pH range of 3.3–3.4. The sensory analysis was carried out when the recognition accuracy of each and every aroma components by the panel was greater than 95%. All the wine samples were analyzed in triplicates with randomized ID.

Please note that we are reporting in this communication results of our computational modeling studies on odor threshold properties of diverse aroma components of wine. We have neither performed experimental aroma component analysis of wine nor determined odor threshold of different components. These values were collected as is from ref. 1 and 23. While preparing the dataset, we have found that the odor threshold values of a few compounds are different in the two references. In cases when the difference is small, we have taken an average value for that particular compound, otherwise (if difference is high) the compound is removed from the main dataset for model development purpose. In that way, we had to remove only four common compounds from the curated data set as recommended by Fourches *et al.*^26^ The original experimental odor threshold data was converted to logarithmic unit (nmol), *i.e.*, log(OT) values spanning from −0.580 to 7.319. The details of the dataset are reported in the ESI Table S1.† Note that a lower value of log(OT) signifies a more potent odorant.

### 2.2 Descriptor calculation

“*The molecular descriptor is the final result of a logic and mathematical procedure which transforms chemical information encoded within a symbolic representation of a molecule into a useful number or the result of some standardized experiments*.”^27^ Geometry optimized molecules were used to calculate a set of both 2D and 3D descriptors using Dragon software version 6 (http://www.talete.mi.it/products/dragon description.htm) (constitutional indices, ring descriptors, connectivity indices, functional group count, atom centered fragments, atom type E-state indices and 2D atom pairs),^28^ PaDEL-Descriptor (http://www.yapcwsoft.com/dd/padeldescriptor) software (extended topochemical atom (ETA) indices descriptors) and Cerius 2 version 4.10 software (structural, electronic and spatial descriptors).^29^ Constant and near constant values (standard deviation less than 0.0001) of the variables, descriptors with at least one missing value, descriptors with all missing values and descriptors with (absolute) pair correlation larger than or equal to 0.95 were excluded from the initial pool of descriptors.

### 2.3 Division of the dataset

The potential of a model to predict the property/activity of the molecules ensures its applicability for the prediction of untested molecules. Thus, splitting of the dataset into training and test sets is crucial to develop a statistically robust QSPR model. In this work, we have employed a clustering technique, “Modified *k*-medoid,”^30^ using a tool developed in our laboratory (http://teqip.jdvu.ac.in/QSAR_Tools/DTCLab) for splitting the dataset into a training set and a test set. Six clusters were generated based on the features available for the respective compounds. We have taken approximately 25% compounds from each cluster for the test set (21 compounds) and remaining 75% compounds for the training set (64 compounds). The QSPR model was developed by using training set compounds and subsequently validated by the test set compounds.

### 2.4 Descriptor selection and model development

After division of the dataset, we have employed various strategies to reduce the size of descriptor pool for development of the final model. Firstly, we have developed a few Genetic Function Approximation (GFA)^31^ models using both linear and spline options in Cerius 2 software. Among the GFA models, we have selected five models based on cross-validated correlation coefficient (*Q*^2^) and predictive *R*^2^ (*R*_pred_^2^). From the selected five models we have selected both common and uncommon descriptors (total 32 descriptors). After that we have run best subset selection for development of six descriptor models (taking only 32 descriptors) using a software developed in our laboratory (http://teqip.jdvu.ac.in/QSAR_Tools/DTCLab). From the developed models obtained after best subset selection, we have taken five models based on MAE based criteria for the test set containing ten descriptors (both common and uncommon). Finally, we have run partial least square regression (PLS) using SIMCA-P software^32^ and developed a PLS model containing seven descriptors.

#### 2.4.1 Genetic function approximation (GFA)

The GFA algorithm evolved from the knowledge of Holland's genetic algorithm (1975)^33^ and Friedman's (1990) multivariate adaptive regression splines (MARS) algorithm.^34^ It was developed from an analogy with the evolution of DNA. Unlike the stepwise regression method, GFA allows to develop multiple models instead of a single model and select the best model based on the fitness and predictive potential of the model as well as mechanistic interpretability of the descriptors. We have used Cerius 2 4.10 version^33^ for the development of GFA models. In GFA, an initial population of equations is built by random selection of descriptors followed by cross over between pairs of those equations. The quality of an individual model was assessed by a fitness function or “Lack of Fit (LOF)” score. The quality of the model improves if the LOF score decreases. After the rating of initial models based on the LOF score, genetic cross-over operation is repeatedly performed. First, two good models are selected as parents and each parent is randomly cut into two pieces, and random cross-over is done between two pieces taking one from each parent, and finally a new model (daughter model) is generated. Good combinations of genes are discovered after many mating step (genetic cross-over) and spread through the population. To run the GFA, we have assigned some options like mutation probabilities (kept at 50% with 5000 iterations), smoothness parameters (kept at 1.00), initial equation length value, *i.e.*, number of descriptors (was set to four) and finally, no fixed length for the final equations. From our previous experiences using several data sets, we have seen that a GFA run with more than 5000 iterations leads to models with either poor predictive ability as evidenced from lower *Q*^2^ or no further improvement of predictive ability than the ones obtained at 5000 iterations. Here, we have used spline terms, designated by angular bracket (〈〉, chevrons), which consider some aspect of nonlinearity. Note that, in this work, we have used GFA algorithm not for development of final QSPR model, but for selection of important descriptors from a large pool of descriptors to reduce the noise in the final model.

#### 2.4.2 Best subset selection

The descriptors (32 descriptors) selected from five GFA models were used for best subset selection using a software tool developed in our laboratory (http://teqip.jdvu.ac.in/QSAR_Tools/DTCLab) in order to optimize the best descriptor combinations among the reduced pool of descriptors which can extract all the requisite structural information responsible to regulate the odor threshold property of the molecules. We have selected the best five multiple linear regression (MLR) models obtained from six descriptors combinations based on the MAE-based criteria^35^ of the validation sets.

#### 2.4.3 Partial least squares (PLS)

Specifically, the PLS technique^36^ is more appropriate in the cases where the matrix of predictors has higher number of variables than observations, and also when there is some intercorrelation among the *X*-variables. A latent variable approach is used to find out the fundamental relations between X-matrix and Y-matrix to model the covariance structures in these two spaces. PLS is normally used in combination with cross-validation to obtain the optimum number of latent variables which ensures that the developed models are selected based on their ability to predict the data rather than to fit the data.^37^ In this work, we have developed the PLS model employing the leave-one-out (LOO) cross-validation method for selection of the number of latent variables.

**Fig. 1:**
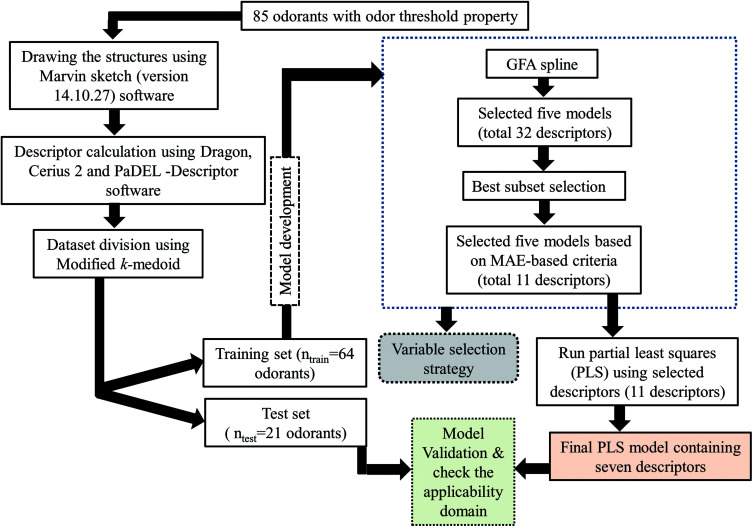
Schematic representation of the steps involved in the development of the final PLS model.

The steps involved to develop the final PLS model is illustrated schematically in Fig. 1.

### 2.5 Validation of the model

In this work, we have checked the statistical quality of the derived models to judge the robustness in terms of fitness, stability and classical predictivity measures using both internal and external validation parameters. We have employed different statistical parameters like determination coefficient (*R*^2^), variance ratio (*F*), standard error of estimate (*s*), adjusted *R*^2^ (*R*_a_^2^) *etc.* But, studies^35^ have shown that these parameters are not sufficient to judge the actual quality and predictive ability of the model. Thus, we have employed some other classical statistical metrics like leave-one-out cross-validated correlation coefficient (*Q*^2^), *R*_pred_^2^, *Q*_F2_^2^ and concordance correlation coefficient (CCC), and calculated different *r*_m_^2^ metrics. Among the above said parameters, *Q*^2^ is used for internal validation, *R*_pred_^2^, *Q*_F2_^2^ and CCC are used for external validation and *r*_m_^2^ metrics are used for both internal (*r*_m(LOO)_^2^ and Δ*r*_m(LOO)_^2^) and external (*r*_m(test)_^2^ and Δ*r*_m(test)_^2^) validation.^38–40^ The threshold value for each of *Q*^2^, *r*_m(LOO)_^2^, *R*_pred_^2^, *Q*_F2_^2^ and *r*_m(test)_^2^ is 0.5, for CCC, this is 0.750 (ref. 41 and 42) and maximum limit for Δ*r*_m(LOO)_^2^ and Δ*r*_m(test)_^2^ parameters is 0.2. Further, Roy *et al.*^35^ proposed mean absolute error (MAE) based criteria for better understanding of the quality of predictions. Here, we have selected the best model based on MAE-based criteria because the *R*^2^ based criteria may sometimes be misleading.^35^ These authors^35^ anticipated that a true indication of prediction quality in terms of prediction errors can be achieved from the error based metrics because they do not judge the performance of the model in comparison with the mean response. The final PLS model was also validated using an additional randomization test^43^ through randomly reordering (100 permutations) the dependent variable (log(OT)) using SIMCA-P software^32^ to make sure that the model was not obtained by chance. In this case, the *Y*-variable values (odor threshold data) are randomly permuted by keeping the X-matrix intact, followed by PLS run. Each and every randomization and consequent PLS run analysis generates a new set of *R*^2^ and *Q*^2^ values. The *R*^2^ and *Q*^2^ values are plotted against the correlation coefficient between the original *Y*-values and the permuted *Y*-values. The PLS model is considered to be valid if the parameter *R*_int_^2^ is less than 0.4 and the parameter *Q*_int_^2^ is less than 0.05. We have also checked the acceptability of the final model using an external validation parameters proposed by Golbraikh and Tropsha.^44^

### 2.6 Applicability domain (AD)

“*The applicability domain of a (Q)SAR is the physico-chemical, structural, or biological space, knowledge or information on which the training set of the model has been developed, and for which it is applicable to make predictions for new compounds. The applicability domain of a (Q)SAR should be described in terms of the most relevant parameters, i.e., usually those that are descriptors of the model. Ideally the (Q)SAR should only be used to make predictions within that domain by interpolation not extrapolation*.” This definition is very helpful for elucidation of the insightful meaning of the “applicability domain” approach. The AD of QSAR model represented by the response and the chemical structure space, is characterized by the molecular properties of the training set compounds only. The developed QSAR model can predict the newly designed compound appropriately when the compound lies within the region of chemical space of the training set molecules. In this work, we have checked the domain of applicability of both the training and test sets compounds employing a DModX (distance to model X) approach^36^ at 99% confidence level using SIMCA-P software.^32^

### 2.7 PCA plot

Factor analysis^45^ was performed using 20 selected training set compounds (10 of which have lowest odor threshold property and remaining 10 show the highest odor threshold property in the training set) employing model descriptor variables, which were to be considered. In this work, the PCA score plot was performed to see whether the developed model is capable to differentiate the molecules bearing higher and lower odor threshold properties or not. The purpose of factor analysis is to exhibit multidimensional data in a space of lower dimensionality with minimum loss of information (explaining >99% of the variance of the data matrix) and to pull out the basic features behind the data with ultimate goal of interpretation and/or prediction. The data matrix is first standardized in factor analysis, and correlation matrix and subsequently reduced correlation matrix are constructed. An eigenvalue problem is then solved and the factor pattern can be obtained from the corresponding eigenvectors. We have used the principal component method to extract the factor. To obtain Thurston's simple structure, the factors were rotated by VARIMAX rotation. Here, we have considered first two factor scores for the PCA score plot that contributed maximum of the variance. In the PCA score plot, the molecular structure is characterized by the property that as many variables as possible fall on the coordinate axes when presented in common factor space, so that largest possible number of factor loadings becomes zero.

### 2.8 Software used

All the chemical structures were drawn using Marvin sketch (version 14.10.27) software (http://www.chemaxon.com/). The molecular descriptors were calculated by using three software namely Dragon version 6 (http://www.talete.mi.it/products/dragon description.htm), Cerius 2 (version 4.0)^29^ and PaDEL-Descriptor (http://www.yapcwsoft.com/dd/padeldescriptor) software. To perform the cluster analysis, we have used modified *k*-medoid (http://teqip.jdvu.ac.in/QSAR_Tools/DTCLab) software. In order to optimize the best descriptors combinations among the descriptors pool, we have run best subset selection using a software developed in our laboratory (http://teqip.jdvu.ac.in/QSAR_Tools/DTCLab). The PLS analysis was performed by using MINITAB Software (version 14.13) (http://www.minitab.com/en-US/default.aspx). SIMCA-P software^32^ was used to perform PLS model randomization, variable importance plot, score plot, regression coefficient plot and loading plot. PCA score plot was developed by using SPSS software.^46^

## 3. Results and discussion

A PLS model (mentioned below in eqn (1)) for odorants was developed using a reduced pool of descriptors obtained from application of various strategies as discussed in Materials and methods section. The statistical quality of the model was assessed by employing different internal and external validation parameters. The results obtained from internal and external validation metrics ensured the acceptable quality in terms of fitness, stability and classical predictivity measures. Recently, Roy *et al.*^35^ showed that *R*^2^ based criteria may be misleading in some cases. They have been proposed mean absolute error (MAE) based criteria for better understanding of the quality of predictions. Using the MAE based judgment, the internal set and external set predictivity was found to be “moderate” and “good” respectively indicating the acceptability of the PLS model. The statistical results obtained from different validation parameters of the PLS model are depicted in Table 1. The results obtained from the Golbraikh and Tropsha criteria (Table 2) showed that the model is acceptable. The randomization results showed that the model is not obtained by any chance as confirmed by *R*_int_^2^ and *Q*_int_^2^ values which are below to the stipulated values (*R*_int_^2^ < 0.4 and *Q*_int_^2^ < 0.05) (Fig. S1†). The proximity of the observed values and the predicted values for the odorants in the dataset can further be ascertained from the scatter plot as shown in Fig. 2.1
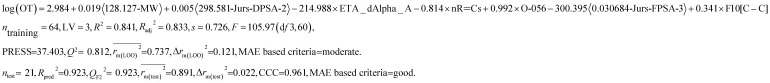


**Table 1:** Statistical quality and validation parameters of the final PLS model developed using both 2D and 3D descriptors

Model type	Descriptors	*R* ^2^	*R*_a_^2^	*Q* ^2^	LV	*s*	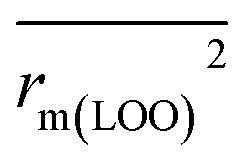	Δ*r*_m(LOO)_^2^	MAE based criteria (training)	*R*_pred_^2^	*Q*_F2_^2^	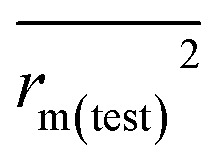	Δ*r*_m(test)_^2^	CCC	MAE based criteria (test)
PLS model with both 2D and 3D descriptors	〈128.127-MW〉, 〈298.581-Jurs-DPSA-2〉, ETA_dAlpha_A, nR=Cs,O-056, 〈0.030684-Jurs-FPSA-3〉, F10[C–C]	0.841	0.833	0.812	3	0.726	0.737	0.121	**Moderate**	0.923	0.923	0.891	0.022	0.961	**Good**

**Table 2:** Results of the final PLS model obtained according to Golbraikh and Tropsha's criteria

	Parameters	Value	Remarks	Threshold value
1	*r* ^2^	0.923	Passed	*r* ^2^ > 0.6
2	[(*r*^2^ − *r*_0_^2^)/*r*^2^]	0.00062	Passed	<0.1
	[(*r*^2^ − *r*_0_′^2^)/*r*^2^]	0.00227	Passed	
3	*k*	1.0015	Passed	0.85 < *k* or *k*′ < 1.15
	*k*′	0.982		

**Fig. 2:**
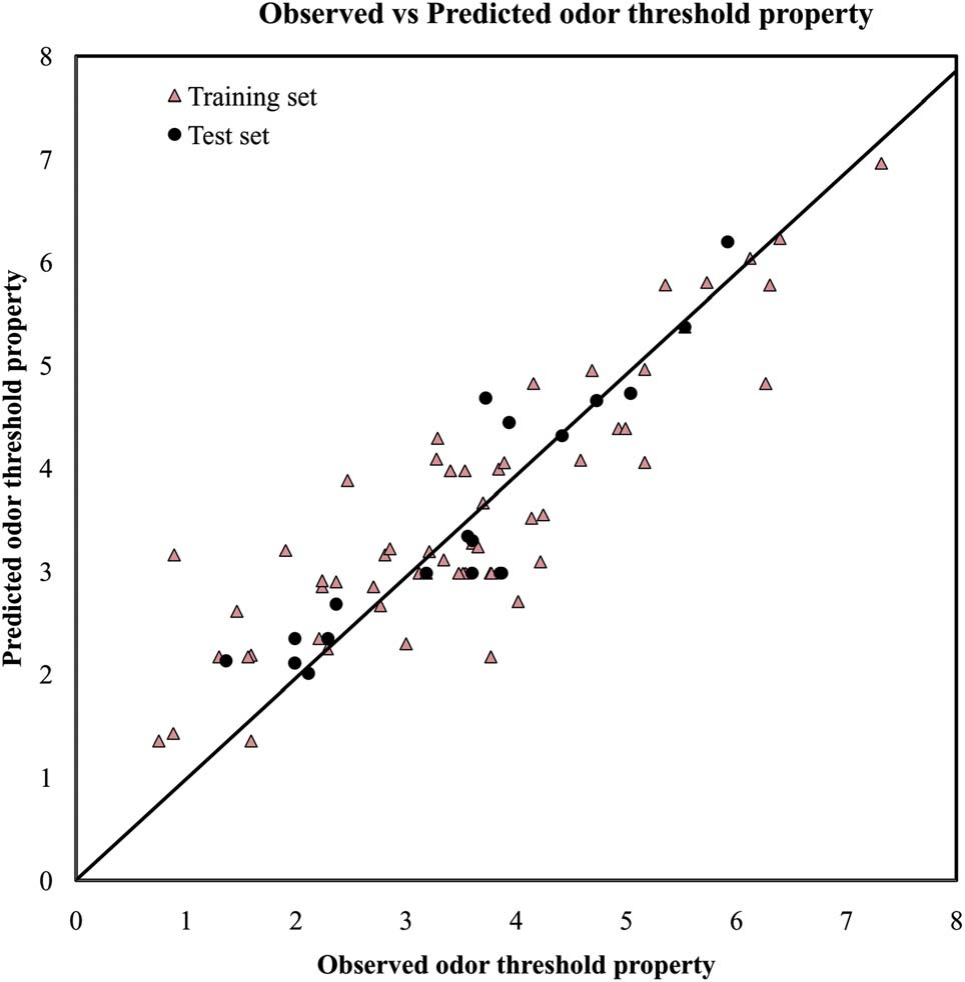
The scatter plot of the observed and the predicted values of odor threshold property for the final PLS model.

The PLS model (eqn (1)) is derived from 3 latent variables. The information obtained from the PLS model suggested that 7 descriptors, as mentioned below, are responsible to regulate the odor threshold of the odorants.

The positive and negative contributions of the descriptors towards the odor threshold property can be easily identified from the regression coefficient plot (Fig. S2†). The descriptors with positive regression coefficients (〈128.127-MW〉, 〈298.581-Jurs-DPSA-2〉, O-056 and F10[C–C]) indicate that the odor threshold property may be raised by increasing the descriptor values while the descriptors bearing negative regression coefficients (ETA_dAlpha_A, nR=Cs and 〈0.030684-Jurs-FPSA-3〉) are inversely correlated with the odor threshold property as discussed below.

The variable importance in projection (VIP)^47^ deals with the influence of individual *X*-variables towards the odor threshold property obtained from the final PLS model. The VIP score can be calculated from the weighted sum of squares of the PLS weights, w*, which take into account the quantity of explained *Y*-variable in every extracted latent variable. The VIP scores give us the idea about the variables which contributed most to the *Y*-variable. Thus, this plot identifies the most and less significant variables towards the odor threshold property. The VIP score of the *j*^th^ variable can be calculated by the following equation.
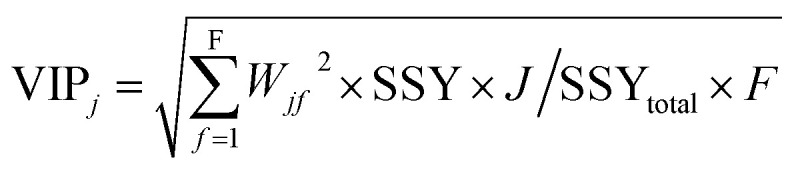


In the above equation, VIP_*j*_ is a measure of the global contribution of *j* variable in the developed PLS model, *W*_*jf*_ is the weighted value for *j*^th^ variable and *f*^th^ component, SSY_*f*_ is the sum of squares of explained variance for the *f*^th^ component and *J* number of *X*-variabless, SSY_total_ is the total sum of squares explained by the dependent variable, *F* is the total number of components and the *w*_*jf*_^2^ gives the importance of the *j*^th^ variable in each *f*^th^ component.

Based on the variable importance plot (VIP) (Fig. S3†), the impact level of the descriptors was found to be in the following order: 〈128.127-MW〉, 〈298.581-Jurs-DPSA-2〉, ETA_dAlpha_A, nR=Cs, O-056, 〈0.030684-Jurs-FPSA-3〉 and F10[C–C].

The highest contribution of the descriptor, MW, reflects its maximum importance for modelling the odorant molecules against odor threshold property. The positive regression coefficient of the spline term 〈128.127-MW 〉 indicates that the value of the molecular weight (MW) should be less than 128.127 for higher odor threshold property. Thus, MW plays a crucial role to alter the odorant property of the molecules. A negative value of the spline term being considered as zero, compounds with lower values of odor threshold can be obtained if the value of MW surpasses the knot of the spline, 128.127. The compounds with low MW may show higher odor threshold property as shown in cases of compounds 2 (isopropyl alcohol), 3 (isobutyl alcohol), 4 (1-butanol), 67 (methanol) and 80 (acetaldehyde) whereas compounds bearing higher MW may show lower odor threshold property as explained by compounds 24 (ethyl 9-decenoate), 25 (isopentyloctanoate), 41 (beta-ionone) and 43 (*trans*-nerolidol). Thus, it can be concluded that for lower odor threshold property, the MW should be more than 128.127.

The second highest significant descriptor, Jurs-DPSA-2, total charge weighted surface area, being present with a spline term 〈298.581-Jurs-DPSA-2〉 bearing a positive regression coefficient, indicates that the numerical value of this descriptor should be more than 298.581 for lower odor threshold property. This descriptor is defined as the total charge weighted positive solvent-accessible surface area (Jurs-PPSA-2) minus total charge weighted negative solvent-accessible surface area (Jurs-PNSA-2). Thus, it may be inferred that there should be some balance between Jurs_PPSA_2 and Jurs_PNSA_2 and the difference of these two parameters should be more than 298.581 for lowering the odor threshold values of the compounds under this study. The compounds with Jurs-DPSA-2 values lower than 298.581 have higher odor threshold property as evidenced by the compounds 2 (isopropyl alcohol), 3 (isobutyl alcohol), 4 (1-butanol), 11 (2,3-butanediol) and 83 (benzyl alcohol) and *vice versa* in case of compounds. 22 (ethyl octanoate), 61 (eugenol), 69 (1-octen-3-ol) and 83 (ethyl cinnamate).

The next highest significant descriptor, ETA_dAlpha_A, the hydrogen bonding propensity and measure of polar surface area parameters, and bearing a negative regression coefficient, implied that the hydrogen bonding propensity and polar surface area of the molecules are influential to lower the odor threshold property as shown in compounds 48 (3-methyl-3-sulfanyl butanol), 50 (3-sulfanylheptanal) and 51 (2-methylsulfanyl-hexanol) and *vice versa* in case of compounds 3 (isobutyl alcohol), 11 (2,3-butanediol) and 82 (benzyl alcohol).

From the above equation, it has been found that the number of aliphatic secondary carbon (sp^2^) atom denoted by nR=Cs plays a crucial role to control the odor threshold property. The negative regression coefficient of this descriptor indicated that the number of double bond attached with an aliphatic carbon atom is suitable for lowering the odor threshold property. It has been observed that compounds bearing aliphatic secondary carbon atom show lower odor threshold property (*e.g.*, compounds 41 (beta-ionone), 43 (*trans*-nerolidol), 45 (furaneol) and 83 (ethyl cinnamate) containing 2, 4, 2 and 2nR=Cs bond respectively) and the compounds without such fragment show higher odor threshold property as shown in compounds 2 (isopropyl alcohol), 4 (1-butanol), 11 (2,3-butanediol) and 82 (benzyl alcohol). Thus, it can be inferred that presence of aliphatic secondary carbon (sp^2^) atom is important to lowering the odor threshold property.

The atom centered fragment descriptor, O-056, indicates the alcoholic fragments present in the molecules. The positive regression coefficient of this descriptor suggests that alcohol functionality is an important property for odorant molecules to enhance the odor threshold property. Thus, the compounds containing alcoholic fragments bear higher odor threshold property as evidenced from the compounds 2, 4, 11 and 67 (all are alcoholic compounds) whereas compounds 22 (ester), 45 (phenolic compound), 47 (furan ring containing ketone) and 61 (phenolic compound) show lower odor threshold property because these compounds are not alcoholic in nature. Thus, it can be interpretated that compounds without any alcoholic fragments may be with lower odor threshold property.

The next significant contribution to the odor threshold property is from Jurs-FPSA-3 descriptor, fractional charged partial surface area, bearing a spline term with a negative regression coefficient. This descriptor is a measure of atomic charge weighted partial positive surface area (Jurs-PPSA-3) and solvent accessible surface area (SASA). Jurs-PPSA-3 is defined as the summation of the products of atomic solvent-accessible surface areas and partial charges of all positively charged atoms. Following the above model, it can be suggested that the numerical value of Jurs-FPSA-3 should be less than 0.030684 to lower the odor threshold property. It has been observed that compounds 11, 46, 54 and 67 showed higher odor threshold property as the numerical values of Jurs-FPSA-3are higher than the knot value of 0.030684 whereas compounds 22, 41 and 43 showed lower range of odor threshold property due to their Jurs-FPSA-3 descriptor value lower than the knot value of 0.030684.
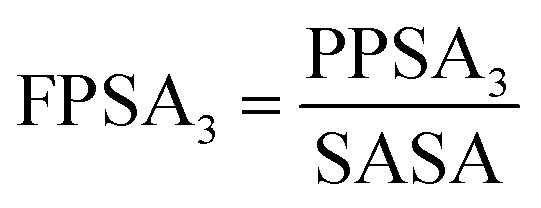


The lowest significant descriptor, F10[C–C], a 2D-atom pair descriptor, indicates the number of [C–C] fragments at a topological distance 10. The positive regression coefficient of this descriptor indicates that the presence of the C–C fragment at the topological distance 10 may enhance the odor threshold property of compounds. A higher number of this fragment correlates to higher odor threshold property of compounds as observed in compounds 72 (ethyl dodecanoate) and 73 (ethyl tetradecanoate) (containing 5, 4 and 6 C–C fragments respectively at topological distance 10) while lower numerical values of this descriptor correlate to lower odor threshold property of odorants as evidenced from compounds 41 (beta-ionone), 43 (*trans*-nerolidol), 50 (3-sulfanylheptanal) and 51 (2-methylsulfanyl-hexanol) (containing no such fragment). Thus, presence of this fragment is not suitable to lower the odor threshold property of odorant molecules.

### 3.1 Score plot of the PLS model

**Fig. 3:**
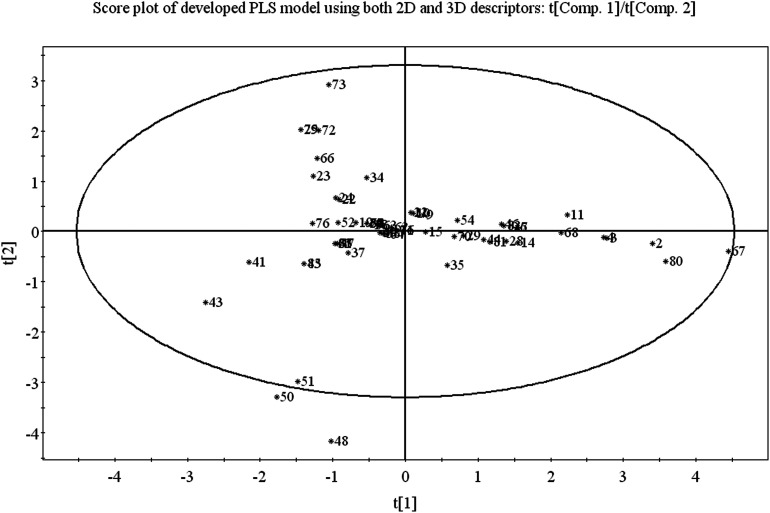
PLS score plot of training set compounds using the developed PLS model.

Score plot deals with the distribution of the compounds in the latent variable space which is defined by the scores. From the score plot, we can identify the similar and dissimilar compounds along with their chemical region in space, *i.e.*, domain of applicability. Here, we have plotted the series of two latent variables (*t*_1_ and *t*_2_) though the model was developed with 3 latent variables. In Fig. 3, the ellipse indicates the applicability domain of the model as defined by Hotelling's *T*^2^. Hotelling's *T*^2^ is a multivariate generalization of Student's *t*-test. The compounds lie close to each other bear similar properties whereas compounds which are situated far apart from each other are said to dissimilar compounds with respect to odor threshold property. As for example, compounds 2 (isopropyl alcohol), 3 (isobutyl alcohol), 4 (1-butanol) and 67 (methanol) are similar compounds and they possess homogeneity while compounds 41 (beta-ionone) and 43 (*trans*-nerolidol) (situated in the left hand side of the plot) are situated far apart from compound nos. 67 (methanol) and 80 (acetaldehyde) (situated in the right hand side of the plot) and represent the heterogeneity with respect to odor threshold property. The compounds which are situated very close to the center (origin) of the plane indicate that they have average properties.

### 3.2 Loading plot of the PLS model

**Fig. 4:**
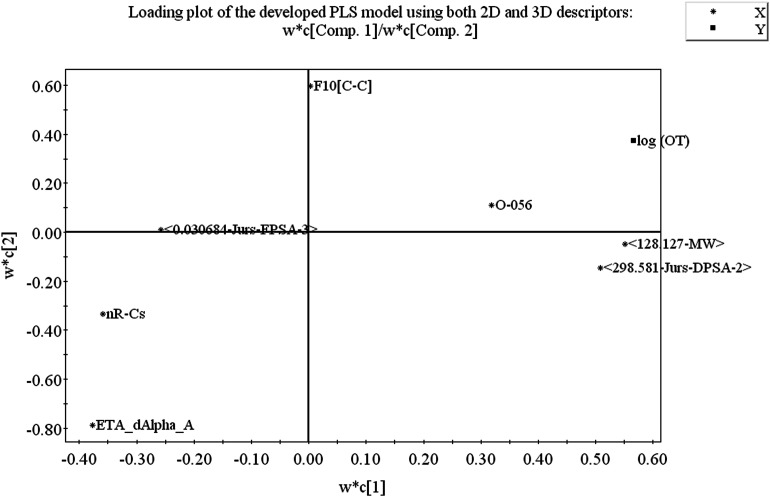
Loading plot of model descriptors and dependent variable (log(OT)).

From the loading plot, we can identify which variables are influential towards the odor threshold property as well as how the variables are correlated. All the variables obtained from PLS model display their relationship in Fig. 4 at the same time. Among the variables, those contributing similar information are grouped together and we can say they are correlated. As for example, the descriptors 〈128.127-MW〉 and 〈298.581-Jurs-DPSA-2〉 are positively correlated, *i.e.*, positioned diagonally in the same quadrant and have similar properties. If the numerical value of one descriptor increases or decreases, there is the same tendency in change of the numerical value of other variable. If the variables are positioned on the opposite side of the plot origin, *i.e.*, diagonally in opposite quadrants, these bear dissimilar properties. From Fig. 4 it is clearly seen that the descriptors O-056 and F10[C–C] are situated oppositely to nR=Cs and ETA_dAlpha_A descriptors and they are inversely correlated, indicating that when the numerical values of O-056 and F10[C–C] increase, the values of nR=Cs and ETA_dAlpha_A decrease and *vice versa*. The impact of the variables can easily be identified from the loading plot. The variables which are situated far from the plot origin, those variables have strong impact on that particular model. Here, 〈128.127-MW〉, 〈298.581-Jurs-DPSA-2〉, ETA_dAlpha_A, nR=Cs and O-056 (situated far away from the origin) have strong impact on the developed PLS model. From the loading plot, we can also identify the weightage of the *X*-variables based on first two components as mentioned in the Table S2.†

### 3.3 Applicability domain

To check the applicability domain of the developed PLS model, we have applied DModX approach (distance to model in X-space) using SIMCA-P software at 99% confidence level. Fig. S4† showed that all the training set compounds are within the critical DModX value (D-critical = 2.222) except two compounds (compounds 43 and 66) which are termed as influential observations. Fig. S5† showed that all the test set compounds are situated within the applicability domain except compound number 74.

### 3.4 PCA plot

We have also performed PCA score plot for selected 20 training set compounds (10 of which have the lowest odor threshold property and 10 of which have the highest odor threshold property) using the model descriptors to check whether the compounds are positioned as clusters in the chemical space or scattered. We have done so to investigate whether the selected descriptors can effectively discriminate the lower odor threshold compounds from those with higher odor threshold. Note that, we have plotted here scores of first two components. From the PCA score plot (Fig. S6†), we have found that lower odor threshold property (compounds 22, 41, 43, 48, 50, 51, 69, 76, 83 and 85) and higher odor threshold property containing compounds (compounds 2, 3, 4, 7, 11, 44, 67, 68, 80 and 82) are distributed into two zones as shown in Fig. S6† as indicated by ellipses. The factor loading values showed that three descriptors namely 〈128.127-MW〉, 〈298.581-Jurs-DPSA-2〉 and ETA_dAlpha_A out of seven descriptors are more discriminating. We have also interpreted from the loading that the score of component one should be low and score of component 2 should be high for lower odor threshold property. Thus, the molecular weight of odorant should be more than 128.127 (as shown in compounds 22, 41, 43, 50, 51, 69, 76, 83 and 85), the numerical value of Jurs-DPSA-2 should be more than 298.581 (as shown in compounds 22, 41, 43, 69, 83 and 85) and ETA_dAlpha_A value should be higher (as shown in compounds 48, 50 and 51) for lowering the odor threshold property whereas compounds like 2, 3, 4, 7, 11, 44, 67, 68, 80 and 82 bear higher odor threshold property due to their molecular weight less than 128.127, Jurs-DPSA-2 value less than 298.581 and the numerical value of ETA_dAlpha_Abeing zero. Thus, the PCA score plot justified the interpretation of the PLS model further.

### 3.5 Prediction of “composite” odor threshold property of various types of wine

In this work, we have also predicted the “composite” odor threshold property of different types of wine namely dry white wine (DW), delay harvested sweet wine (SW), noble rot wine after 1^st^ and 2^nd^ pressing (NR1 and NR2 respectively) using the information (model descriptors) obtained from the developed PLS model. Here, we have tried to justify whether the model is capable of predicting the “composite” odor threshold property of different components present in the different types of wine or not. For the calculation of “composite” odor threshold property of each class of wine, we have calculated the “composite” value of each descriptor obtained from the PLS model (total seven descriptors) by taking into account the different components present in each type of wine. Here, for calculation of “composite” descriptor values, we have taken 42 components for DW wine, 42 components for SW wine, 45 components for NR1 wine and 45 components for NR2 wine. Finally, the “composite” descriptor values are put into the developed PLS model, and the “composite” odor threshold property of each type of wine is calculated. The steps involved in the calculation of “composite” value of each descriptor are depicted in Table S3.† The results as depicted in Table S4† showed that the developed PLS model can predict the “composite” odor threshold property of different types of wine. Based on the developed model, the order of “composite” odor threshold property (lowest to highest) obtained from different wines are (1) NR2 (log(OT) = 4.566), (2) NR1 (log(OT) = 4.580), (3) SW (log(OT) = 4.819) (4) DW (log(OT) = 5.169) which are well corroborated with the observations reported by Wang *et al.*^1^ that artificial noble rot wines are richer in aroma compounds than dry white wine and delay harvested sweet wine.

## 4. Conclusions

The aim of this work was to determine the quantitative relationship between the odor threshold property (log(OT)) of chemically diverse molecules present in wines and their structural properties employing the regression-based modeling technique. A variable selection approach was applied to reduce the initial pool of descriptors which proved to be an efficient approach to extract the influential descriptors for development of the final model. The final PLS model showed good results based on all statistical quality and validation metrics. This model was developed in compliance with the OECD principles. Based on the insights obtained from the developed PLS model, we have found that molecular weight, total charge weighted surface area, hydrogen bonding propensity and polar surface area, presence or absence of aliphatic secondary carbon (sp^2^), presence or absence of alcoholic fragments, fractional charged partial surface area and the [C–C] fragment at topological distance 10 play a crucial role to influence the odor threshold property of molecules. Thus, it can be concluded that to lower the odor threshold properties of odorant compounds present in different wines, (i) the molecular weight of the odorant molecules should be more than 128.127; (ii) total charge weighted surface area should be high; (iii) hydrogen bonding propensity and polar surface area should be more; (iv) the molecules should bear aliphatic secondary carbon (sp^2^); (v) the alcoholic fragments should be absent; (vi) fractional charged partial surface area should be low; and (vii) the molecules should not contain [C–C] fragment at topological distance 10. We have also concluded that the developed model can predict the “composite” odor threshold property of various types of wine which is well corroborated with the previously reported observation.^1^ The developed model can be successfully utilized for *in silico* prediction of odor threshold values of diverse classes of compounds if they fall within the AD of the developed PLS model.

## Conflicts of interest

There are no conflicts to declare.

## Supplementary Material

RA-008-C7RA12295K-s001
