# Supplementary material for: Chemometric modeling of odor threshold property of diverse aroma components of wine
Source: RSC Adv. 2018 Jan 25;8(9):4750–60. doi: 10.1039/c7ra12295k (PMC9092618; doi:10.1039/c7ra12295k)
Supplement: RA-008-C7RA12295K-s001 [file RA-008-C7RA12295K-s001.pdf]

This journal is © The Royal Society of Chemistry 2018

Table S1

List of aroma components with observed and model derived odor threshold property

Sl. No.	NAME	observed odor threshold property (log (OT))	Predicted odor threshold property
1*	1-propanol	5.920	6.198
2	isopropyl alcohol	6.397	6.231
3	isobutyl alcohol	5.732	5.804
4	1-butanol	6.306	5.778
5*	isopentyl alcohol	5.532	5.374
6	isohexyl alcohol	4.690	4.950
7	3-methyl- pentan-1-ol	5.167	4.962
8*	1-heptanol	3.935	4.446
9	2-ethyl-1-hexanol	4.584	4.078
10	1-octanol	3.839	3.992
11	2,3-butanediol	6.124	6.038
12	1-decanol	3.403	3.976
13*	dodecan-1-ol	4.730	4.658
14	ethyl acetate	4.930	4.386
15	isobutyl acetate	4.139	3.518
16	ethyl butanoate	2.236	2.851
17*	ethyl isovalerate	1.362	2.131
18	isopentyl acetate	2.362	2.900
19	hexyl acetate	4.017	2.707
20*	ethyl hexanoate	1.987	2.109
21	ethyl 2-hydroxy-3-methylbutyrate	3.534	3.976
22	ethyl octanoate	1.463	2.612
23	ethyl decanoate	2.999	2.296
24	ethyl 9-decenoate	2.703	2.852
25	isopentyl octanoate	2.766	2.666
26*	propanoic acid	5.039	4.730
27*	isobutyric acid	4.417	4.317
28	butyric acid	3.285	4.291
29	isopentanoic acid	2.468	3.881
30*	hexanoic acid	3.558	3.343
31	octanoic acid	3.540	2.984
32	decanoic acid	3.764	2.984
33	9-decenoic acid	3.769	2.170
34	dodecanoic acid	3.698	3.666
35	(E)-3-hexen-1-ol	3.601	3.275
36*	(Z)-3-hexen-1-ol	3.601	3.300

37	linalool	2.210	2.348
38	ALPHA-terpineol	3.210	3.193
39	citronellol	2.806	3.162
40*	trans-geraniol	2.289	2.348
41	BETA-ionone	-0.330	-0.046
42*	ALPHA-ionone	-0.330	-0.410
43	Beta-damascenone	-0.580	-1.178
44	furfural	5.167	4.056
45	furaneol	1.591	1.356
46	furfuryl alcohol	4.158	4.825
47	3-hydroxy-4,5-dimethylfuran-2(5H)-one	1.591	2.186
48	3-methyl-3-sulfanyl butanol	-0.381	-0.531
49*	3-sulfanylhexanol	-0.350	-0.302
50	3-sulfanylheptanal	-0.262	-0.774
51	2-methylsulfanyl-hexanol	-0.268	0.024
52	Gama-nonalactone	2.283	2.247
53*	phenyl acetic acid	3.866	2.984
54	2-phenyl-ethanol	4.992	4.385
55*	phenethyl acetate	3.183	2.984
56*	ethyl phenylacetate	3.597	2.984
57	methyl salicylate	3.517	2.984
58	ethyl 4-hydroxybenzoate	3.478	2.984
59	vanillin	3.119	2.984
60	ethyl vanillin	3.775	2.984
61	eugenol	1.563	2.170
62	guaiacol	1.906	3.206
63	thymol	4.221	3.094
64	alpha-Terpineol	2.853	3.221
65*	Nerol	1.988	2.348
66	cis,trans-Farnesol	3.653	3.239
67	Methanol	7.319	6.964
68	Isoamyl alcohol	5.532	5.374
69	1-Octen-3-ol	0.892	3.162
70	Ethyl propanoate	4.246	3.552
71	Ethyl butyrate	2.236	2.909
72	Ethyl dodecanoate	3.340	3.113
73	Ethyl tetradecanoate	3.892	4.054

74*	Ethyl hexadecanoate	3.722	4.683
75*	Ethyl isobutyrate	2.111	2.010
76	Ethyl 2-methylbutyrate	0.885	1.430
77*	Isoamyl acetate	2.362	2.683
78	2-Phenylethyl acetate	3.183	2.984
79	Isoamyl octanoate	2.766	2.666
80	Acetaldehyde	5.356	5.779
81	Benzaldehyde	3.275	4.088
82	Benzyl alcohol	6.267	4.823
83	Ethyl cinnamate	0.754	1.356
84*	Ethyl 2-furoate	3.853	2.984
85	Phenol, 4-ethenyl-2-methoxy-	1.300	2.170

*denoted test set compounds

Table S2

Weightage of descriptors for first two components.

Descriptors	Weightage based on the first two components	
	Component 1	Component 2
nR=Cs	-0.358618	-0.332316
O-056	0.318237	0.111099
F10[C-C]	0.00186353	0.596138
ETA_dAlpha	-0.376946	-0.787048
<0.030684-Jurs-FPSA-3>	-0.257966	0.0107757
<128.127-MW>	0.550793	-0.0489371
<298.581-Jurs-DPSA-2>	0.508043	-0.145935

Table S3
The steps involved in the calculation of "composite" value of each descriptor.

Sl no	OT (nmol)	Fraction	Des 1*fraction	Des n*fraction
1	P1	P1/W1	d1*(P1/W1)	dn*(P1/W1)
2	P2	P2/W1	d1*(P2/W1)	dn*(P2/W1)
.	.	.	.	.
.	.	.	.	.
n	Pn	Pn/W1	d1*(Pn/W1)	dn*(Pn/W1)
	Sum=W1		Sum= composite value of descriptor 1	Sum= composite value of descriptor n

Table S4

Results of the "composite" odor threshold property of different wines obtained from the PLS model.

Types of wine	DW	SW	NR1	NR2
Log(OT)	5.169	4.819	4.580	4.566

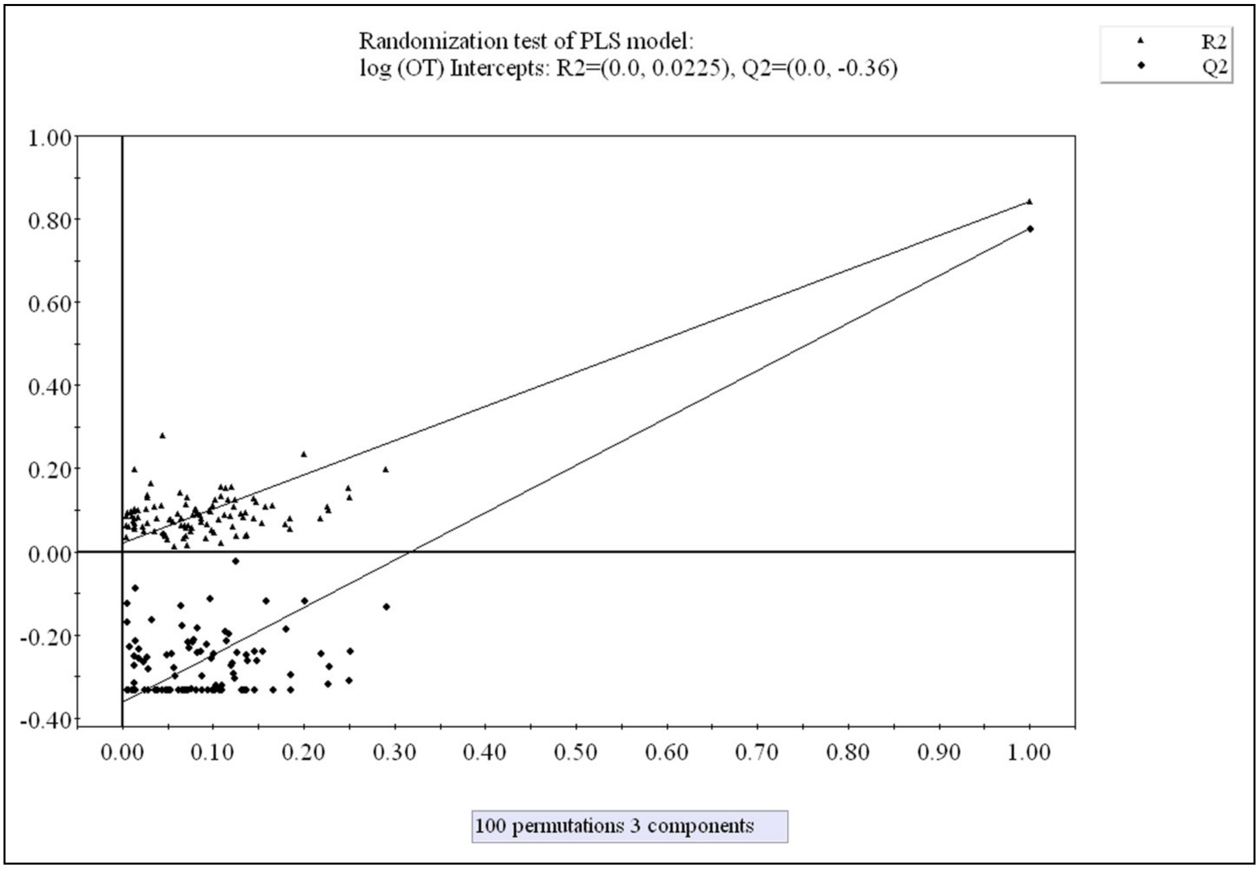

Fig. S1. The randomization plot for the QSPR model derived from PLS analysis.

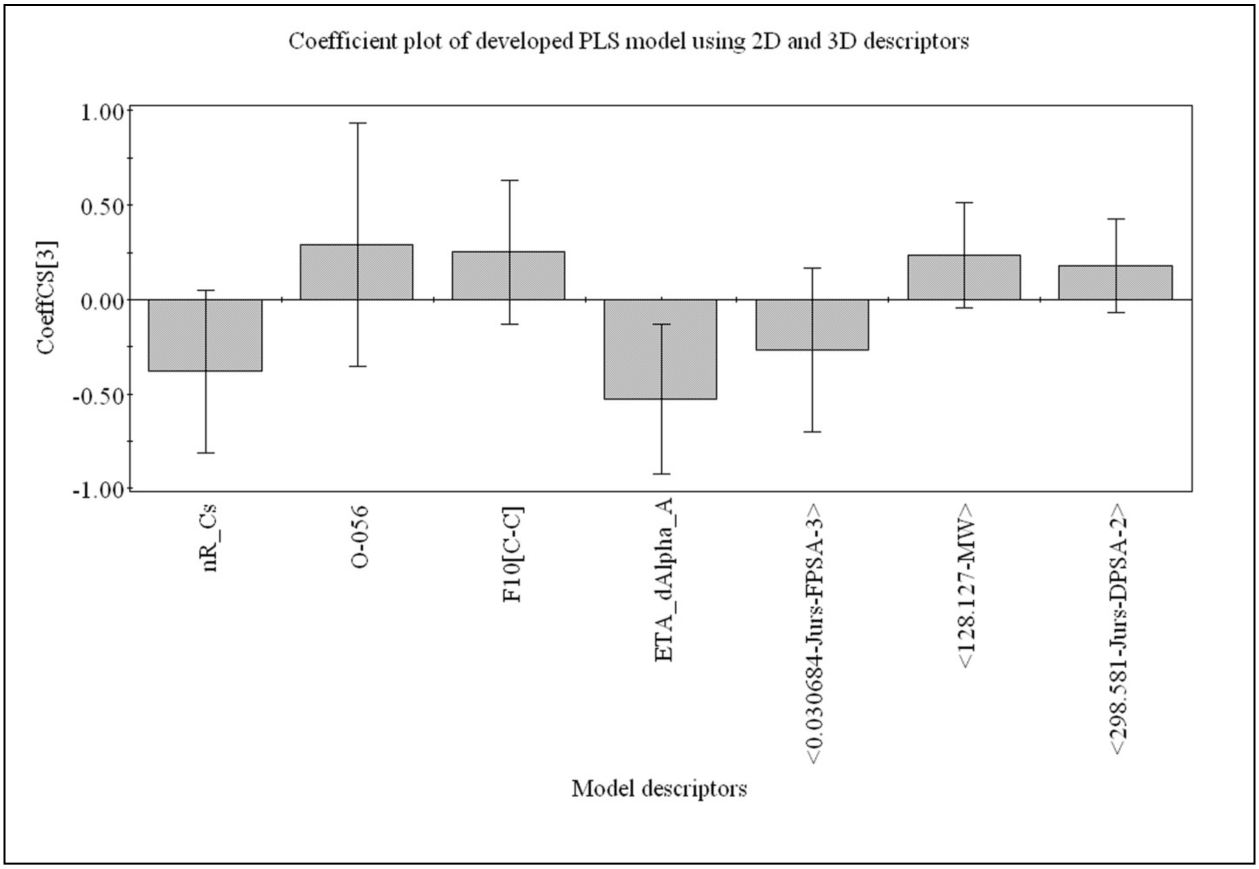

Fig. S2. Regression coefficient plot of the final PLS model.

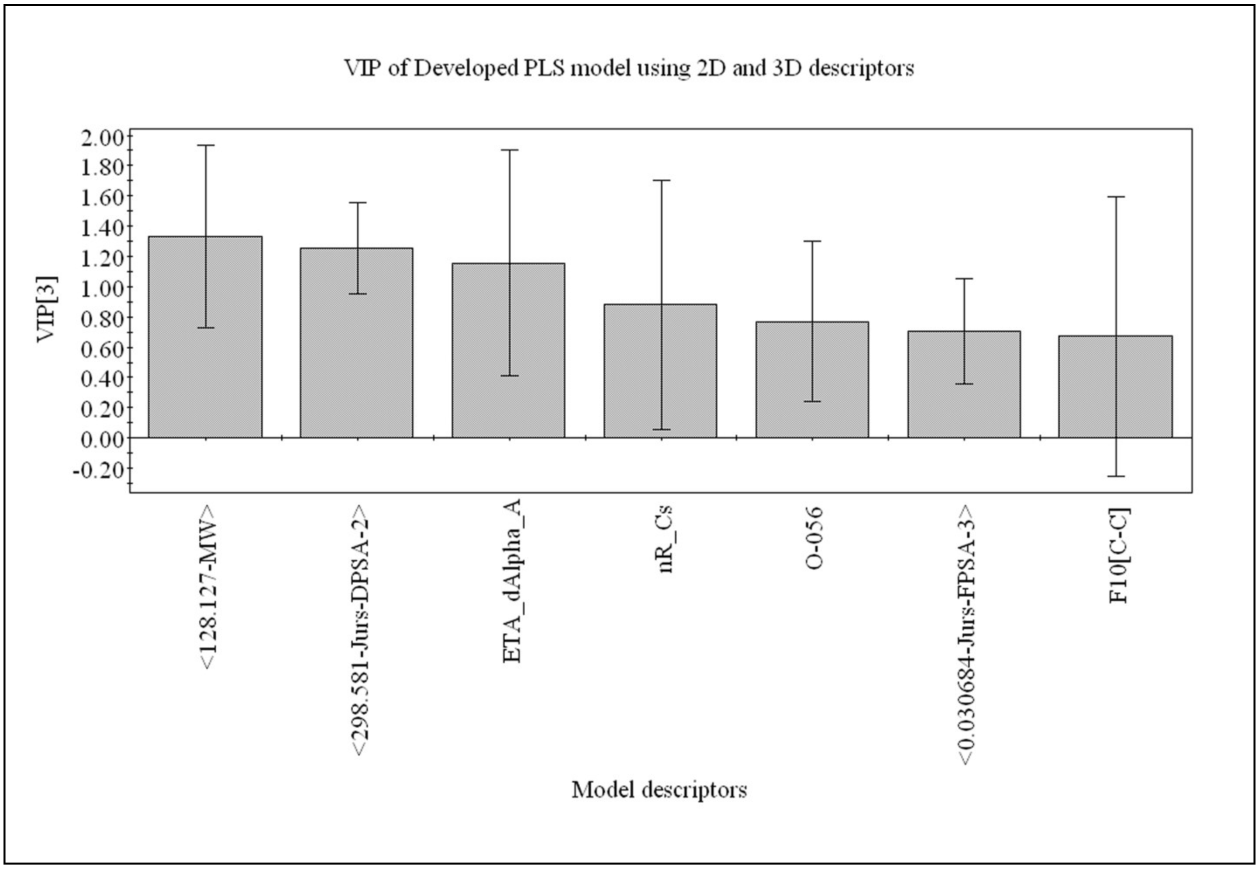

Fig. S3. Variable importance plot (VIP) for the final PLS model.

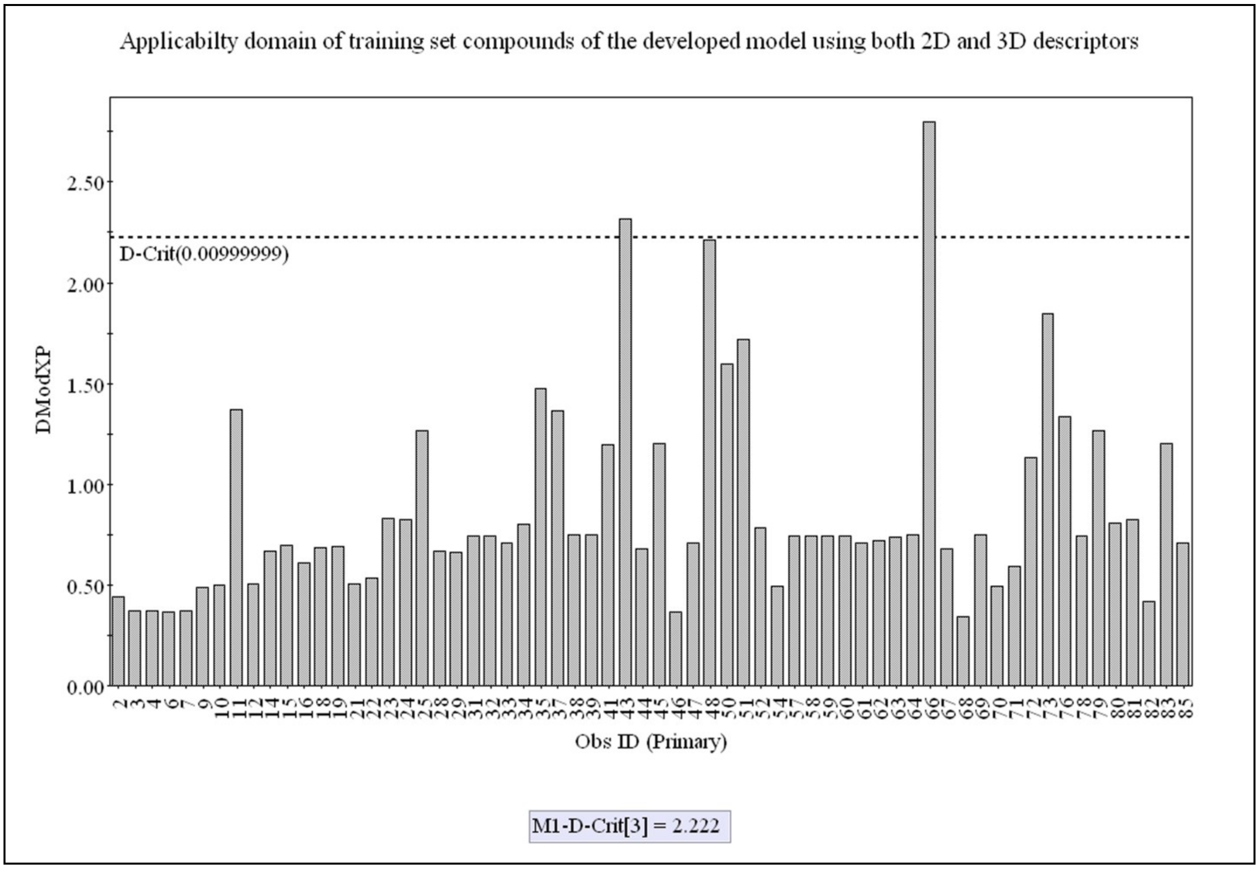

Fig. S4. DModX values of the training set compounds at 99% significance level for the developed model. The dash horizontal line signifies the critical DModX value (2.222) at the 99% confidence level.

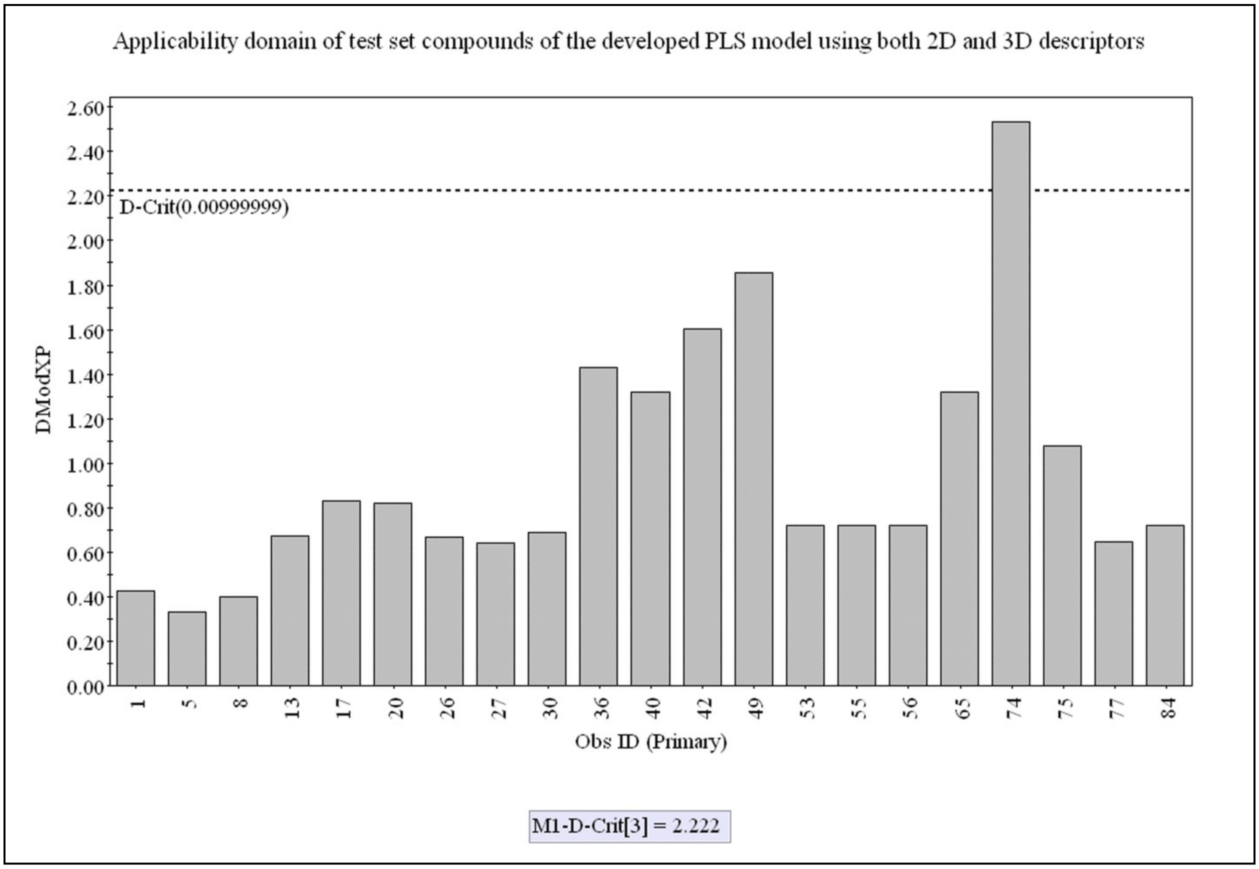

Fig. S5. DModX values of the test set compounds at 99% significance level for the developed model. The dash horizontal line signifies the critical DModX value (2.222) at the 99% confidence level.

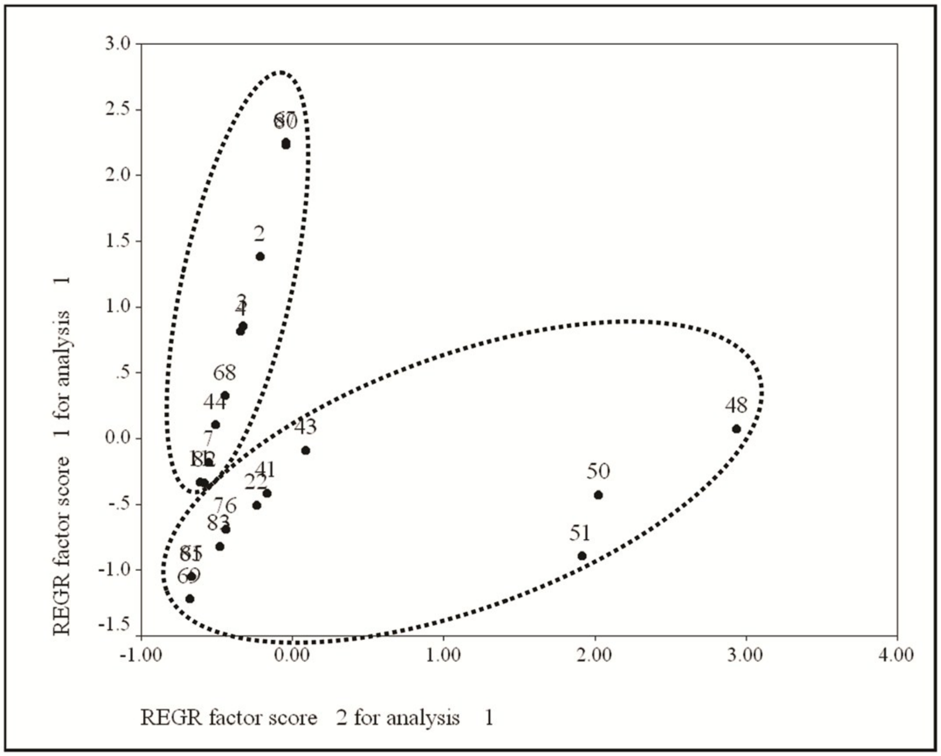

Fig. S6. PCA score plot for selected 20 training set compounds (10 of which have the lowest odor threshold property and 10 of which have the highest odor threshold property) using the model descriptors.
